# Identifying prognostic genes related PANoptosis in lung adenocarcinoma and developing prediction model based on bioinformatics analysis

**DOI:** 10.1038/s41598-023-45005-6

**Published:** 2023-10-20

**Authors:** Chi Zhang, Jiangnan Xia, Xiujuan Liu, Zexing Li, Tangke Gao, Tian Zhou, Kaiwen Hu

**Affiliations:** 1 Oncology Department, Dongfang Hospital, Beijing University of Chinese Medicine, Beijing, China; 2 College of Pharmacy, Hunan Traditional Chinese Medical College, Zhuzhou, China

**Keywords:** Prognostic markers, Data mining

## Abstract

Cell death-related genes indicate prognosis in cancer patients. PANoptosis is a newly observed form of cell death that researchers have linked to cancer cell death and antitumor immunity. Even so, its significance in lung adenocarcinomas (LUADs) has yet to be elucidated. We extracted and analyzed data on mRNA gene expression and clinical information from public databases in a systematic manner. These data were utilized to construct a reliable risk prediction model for six regulators of PANoptosis. The Gene Expression Omnibus (GEO) database validated six genes with risk characteristics. The prognosis of LUAD patients could be accurately estimated by the six-gene-based model: NLR family CARD domain-containing protein 4 (*NLRC4*)*,* FAS-associated death domain protein (*FADD*)*,* Tumor necrosis factor receptor type 1-associated DEATH domain protein (*TRADD*)*,* Receptor-interacting serine/threonine-protein kinase 1 (*RIPK1*)*,* Proline-serine-threonine phosphatase-interacting protein 2 (*PSTPIP2*), and Mixed lineage kinase domain-like protein (*MLKL*). Group of higher risk and Cluster 2 indicated a poor prognosis as well as the reduced expression of immune infiltrate molecules and human leukocyte antigen. Distinct expression of PANoptosis-related genes (PRGs) in lung cancer cells was verified using quantitative reverse transcription polymerase chain reaction (qRT-PCR). Furthermore, we evaluated the relationship between PRGs and somatic mutations, tumor immune dysfunction exclusion, tumor stemness indices, and immune infiltration. Using the risk signature, we conducted analyses including nomogram construction, stratification, prediction of small-molecule drug response, somatic mutations, and chemotherapeutic response.

## Introduction

A malignant tumor of the bronchial mucosal epithelium and mucous glands is known as lung cancer, accounting for approximately 2.2 million new diagnoses and 1.8 million mortalities worldwide in 2020^1^. Lung cancer is divided into small-cell lung cancer (SCLC, 15%) and non-small cell lung cancer (NSCLC, 75%) depending on the sort of cell^2^. NSCLC can be classified into three types based on histological categorization: lung squamous cell carcinoma (LUSC), large cell carcinoma, and lung adenocarcinoma (LUAD)^3^. LUAD accounts for approximately 50% of all cases of lung cancer, and the majority of LUAD cases are diagnosed at advanced stages^4^. Treatment options for these patients may include surgery, targeted therapy, radiation, immunotherapy, and chemotherapy. However, the survival rate for LUAD patients beyond 5 years is only 15%^5^. Moreover, this treatment regimen is associated with an increased therapy-related toxicity and risk of surgery, and patients with advanced LUAD continue to be at risk for bad clinical outcomes, and patients with aggressive or advanced LUAD still face adverse clinical outcomes. Consequently, the task at hand is to develop a method for identifying patients who might profit from aggressive therapies. As such, innovative diagnostic biomarkers for prognosis and treatment response in LUAD patients are imperatively necessary.

PANoptosis is a recently discovered type of programmed cell death (PCD), involving the coordinated release of biochemical signals from three PCD pathways—necroptosis, apoptosis, and pyroptosis, and. It has been observed in several cancers, sterile injuries, and infectious diseases^6^. Moreover, PANoptosis is an inflammatory PCD pathway modulated by the PANoptosome complex, whose essential characteristics cannot be explained by necroptosis, apoptosis, or pyroptosis alone. In response to pathogenic factors, specific receptors, like the activated Z-DNA binding protein 1 (ZBP1), initiate the formation of the PANoptosome complex. The Zα domains of ZBP1 identify associated nucleic acids in cells and trigger the NOD-like receptor thermal protein domain associated protein 3 (NLRP3) inflammasome, caspase 8 (CASP8), RIPK3, CASP1, and RIPK1. Molecules required for downstream PCD effectors (i.e., gasdermins, and CASP3/7) are then activated, and PANoptosis is executed by engaging members of the pyroptotic, apoptotic, and necroptotic pathways, leading to lytic inflammatory cell death^7^. Several studies show that PANoptosis plays a crucial function in the antitumor defenses of the body. Given that PANoptosis involves multiple proteins and has tandem properties, promoting PANoptosis can lead to inflammatory cell death, stimulate the immune response, and reduce the likelihood of developing acquired drug resistance^8^. Karki^9^ found that Adenosine deaminase RNA specific-1 (ADAR-1) suppresses PANoptosis by reacting with the Zα2 domain of ZBP1, thereby preventing interactions between ZBP1 and RIPK3. Mice lacking ADAR1 are resistant to melanoma and colorectal cancer. In addition, removal of the ZBP1 Zα2 domain improves tumor development in these mice. Mice lacking interferon regulatory factor 1 (IRF1) are hypersusceptible to development of colorectal cancer owing to defective PANoptosis^10^. Recently, researchers constructed clinical prognostic models for gastric and colon cancers on the basis of PRGs^11,12^.

There has been limited investigation into the expression pattern, predictive value, and molecular function of PRGs in LUAD. Therefore, this study aims to examine the differential expression of PRGs in normal and LUAD samples, and develop a prognostic tool to assess the potential role of PRGs in LUAD. Given the increasing use of immunotherapy and personalized medicine in clinical practice, immune infiltrations have become crucial prognostic indicators for various malignancies. Consequently, our objective is to predict the immune landscape, tumor microenvironment biomarkers, somatic mutations, and drug sensitivities of patients stratified by their risk levels, and provide novel therapeutic options for LUAD patients.

## Material and methods

### Collection of RNA-sequencing transcriptomic data

The Cancer Genome Atlas (TCGA, https://portal.gdc.cancer.gov/) was consulted for RNA-sequencing transcriptomic data and related clinical data^13^. The data involved 412 LUAD cases and 43 adjacent normal samples. Clinical data was collected, including age, gender, stage, and tumor, node, and metastasis (TNM) stage (Table S1). We identified 38 PRGs by screening the GeneCards database (genes with a relevance score > 0.4 were selected, https://www.genecards.org/)^14^ and through literature investigation^11,12, 15–20^. We extracted the expression data for 38 PRGs for subsequent analyses from the TCGA’s LUAD cohort. As validation sets, we used three datasets from the Gene Expression Omnibus database (GEO, http://www.ncbi.nlm.nih.gov/geo)^21^: GSE30219, GSE31201, and GSE50081. Table S2 provides all gene expression matrix used in this study and Table S3 provides a list of the analyzed genes.

### Identification of differentially expressed PANoptosis-related genes in LUAD

Using the Wilcoxon test in R (version R 4.1.2)^22^, differentially expressed PRGs between LUAD and adjacent normal tissues were identified. Significance standards were as follows: false discovery rate (FDR) < 0.05 and absolute |log_2_FC|> 1. Using the "vioplot" R package^23^, volcano graphs were created to illustrate the differential expression of PRGs in LUAD and adjacent normal tissue samples. To determine the relationships between the PRGs, we conducted Spearman's correlation analysis. We used the the Search tool for the retrieval of interacting genes/proteins database (https://string-db.org/)^24^ to query interactions of PANoptosis-related proteins. Interactions with a combined score greater than 0.7 were considered significant, and PRGs with stronger interaction strength were identified as key genes. Subsequently, we constructed a protein–protein interaction (PPI) network. To identify hub modules and genes, we utilized the "cytohubba" and "MCODE" plugins in Cytoscape 3.9.1^25,26^. To functionally annotate PRGs, Gene Ontology (GO) and Kyoto Encyclopedia of Genes and Genomes (KEGG) analyses were done using the “ClusterProfiler” R package (version3.0.4, https://rdocumentation.org/packages/clusterProfiler/versions/3.0.4), where *P*-value < 0.05 represents a statistically significant difference, and the result was visualized by “ggplot2” R package^27^.

### Consensus clustering

Univariate Cox regression analysis was used to screened out prognosis-related PRGs. R package “ConsensusClusterPlus”^28^ was used to sort the LUAD cohort into two distinct subgroups. On the basis of the Kaplan–Meier analysis results, survival curves were generated to compare the overall survival (OS) between groups. Between-group differences in clinical information (i.e., stage, gender, survival status, and age) were detected using the chi-square test.

### Evaluation and verification of the prognostic significance

We estimated the relationship between PRGs and OS using univariate Cox regression analysis. Using the R package “glmnet,”^29^ the least absolute shrinkage and selection operator (LASSO) regression model was used to reduce the number of candidate genes and create the predictive model. In the end, six genes and their coefficients were kept, and we used the minimum criteria to determine the penalty parameter (λ). To calculate the risk score, we multiply the gene expression obtained from the LASSO Cox regression by its coefficients. The median risk score was utilized to categorize LUAD patients into low- and high-risk groups. To conform the usability of this prognostic signature, we compared the OS between the two subgroups using Kaplan–Meier analysis and used the “survival,” “survminer,” and “timeROC” R packages^30–32^ to conduct the receiver operating characteristic (ROC) curve analysis. To evaluate and illustrate the differences in clinically relevant factors among the different risk groups, the chi-square test was employed. Heatmaps were utilized as visual representations. Additionally, three GEO datasets (GSE50081, GSE31201, and GSE30219) were employed for the validation of prognostic signatures.

Univariate and multivariate Cox regression analyses were carried out using the R packages "survivalROC"^33^ and "survival" to determine whether the risk score served as an independent prognostic indicator. The risk scores and stage were used to develop prognostic nomograms for predicting overall survival (OS) in patients with lung adenocarcinoma (LUAD), employing the "rms"^34^ R package. Using calibration diagrams, the congruence between the predicted and actual 1-, 2-, 3-, and 5-year survival probabilities was evaluated.

### Construction of prediction nomogram

Based on the R package “rms”, clinically relevant factors (histological grade, sex, stage, and age) and risk scores were used to construct prognostic nomograms to predict OS in LUAD patients.

### Gene set enrichment analysis

To identify potential mechanisms, we employed Gene Set Enrichment Analysis (GSEA)^35^, analyzing enriched pathways in the high-risk group. The reference gene sets encompassed c2kegg, hallmark, and c5go. Normalized enrichment score > 1, nominal *P* value < 0.05, and FDR *q*-value < 0.25 were the screening conditions.

### Immune landscape analysis

Immune component profiles were evaluated using the TIMER^36^, quanTIseq ^37^, CIBERSORT^38^, xCell^39^, MCPcounter^40^, and EPIC^41^ algorithms, and the "pheatmap" R package^42^ was employed for visualization. We used single-sample GSEA (ssGSEA)^43^ to compute the score of the immune function and infiltration of immune cell subsets. Using the proportion of immune and stromal cells, the ESTIMATE algorithm^44^ computed the scores of immune, stromal, and the tumor purity. We also compared the expression of Major histocompatibility complex (MHC) molecules based on the cluster analysis and signature. Boxplots were utilized to illustrate the differential expression of common immune checkpoints between subgroups, including the T-cell immune receptor with immunoglobulin and immunoreceptor tyrosine-based inhibitory motif domains, programmed death 1 (PD-1), programmed death-ligand 1 (PD-L1), cytotoxic T lymphocyte-associated protein 4 (CTLA4), and tumor necrosis factor receptor superfamily. The tumor immune dysfunction and exclusion (TIDE) index is predictive of a patient's immune checkpoint inhibitor treatment; therefore, we calculated the TIDE score for patients with LUAD in the TCGA using the TIDE database^45^.* P* value < 0.05 was considered to indicate the existence of statistical differences in the above indicators between the different groups.

### Analysis of malignancy characteristics in different risk groups

The angiogenic activity, tumorigenic cytokines, mesenchymal-epithelial-mesenchymal transition (EMT), and stemness scores play crucial roles in determining malignant tumor characteristics^46^. To quantify these indicators for each tumor sample, we utilized the ssGSEA algorithm. We obtained tumor stemness indices (TSIs) for patients with LUAD from a previous study^47^. TSIs were linked to a higher degree of tumor dedifferentiation and tumor stem cells. Somatic mutation information was extracted from the TCGA database, and gene mutation analysis was conducted using the "maftools" package^48^. We assessed the tumor mutation burden (TMB) for each patient and compared it between the two risk groups. A survival analysis was conducted based on the TMB score. Somatic mutations for selected genes in the signature were displayed using the cBioPortal for Cancer Genomics database (http://www.cbioportal.org/)^49^.

### Analysis of drug susceptibility and prediction of small-molecule compounds

The R package “limma”^50^ was utilized to identify differentially expressed genes between the high-and low-risk groups for subsequent small-molecule drug screens. We input the gene list into the connectivity map (CMap, https://clue.io/)^51^ to explore compounds potentially related to the six gene therapies. The connectivity map contains gene expression signatures collected from nine cancer cell lines treated with 2,429 compounds with detailed annotations. We calculated connectivity scores by matching CMap data with six gene signatures, and these scores were found to be inversely correlated with the therapeutic effects of the compounds. The half maximal inhibitory concentration values were predicted for standard chemotherapeutics in the risk subgroups using the R package “pRRophetic.”^52^ The three-dimensional (3D) structures for these candidate drugs were acquired from the PubChem database (https://pubchem.ncbi.nlm.nih.gov/)^53^.

### Immunochemistry validation based on the HPA database

Immunohistochemistry data from the Human Protein Atlas (HPA, http://www.proteinatlas.org/)^54^ were utilized to verify the protein expression level of six prognosis-relevant genes between LUAD and normal lung samples in the TCGA cohort.

### Cell culture and real-time polymerase chain reaction

The lung cell lines A549 (CCL-185™) and H1975 (CRL-5908™), H460 (HTB-177TM) and the normal human bronchial epithelial cell line BEAS-2B (CRL-9609 ™) were acquired from the American Type Culture Collection. The cells were cultured in RPMI 1640 medium (Gibco, C11875500BT). The media were supplemented with 10% heat-inactivated fetal bovine serum (Gibco, 10099-141) and 1% penicillin–streptomycin (Gibco, 15070063). The cells were kept in an incubator at 37 °C and 5% CO_2_. We extracted total RNA from the three cell lines using TRIzol™ reagent (Ambion, 15596-026). cDNA was produced using a cDNA reverse transcription kit (Thermo Fisher Scientific, EP0751). The cDNA was used as the template, and glyceraldehyde 3-phosphate dehydrogenase was used as the internal reference for quantitative reverse transcription polymerase chain reaction (qRT-PCR). According to the manufacturer's guidelines (Yeasen Biotechnology, 10222ES60), a standard two-step PCR amplification procedure was performed. The relative expression of the genes was calculated using the 2^−ΔΔCT^ method; the primers are listed in Table S4.

### Statistical methods

Statistical analysis was performed using R software (version 4.2.1). Statistical significance was set at a p-value < 0.05 and FDR < 0.05. The PRGs signature was constructed using the LASSO-Cox regression model. Gene expression, tumor-infiltrating immune cells, immune checkpoints, and immune function were analyzed using the paired Student's t-test or Wilcoxon test. The OS of the groups was compared using Kaplan–Meier analysis. The predictive performance of the model was evaluated using time-dependent ROC analysis. Spearman correlation analysis was conducted to assess the correlation between the risk score and immune cell infiltration. Cox regression analysis was used to determine independent predictors.

## Results

### Identification of differentially expressed PRGs in LUAD

**Figure 1:**
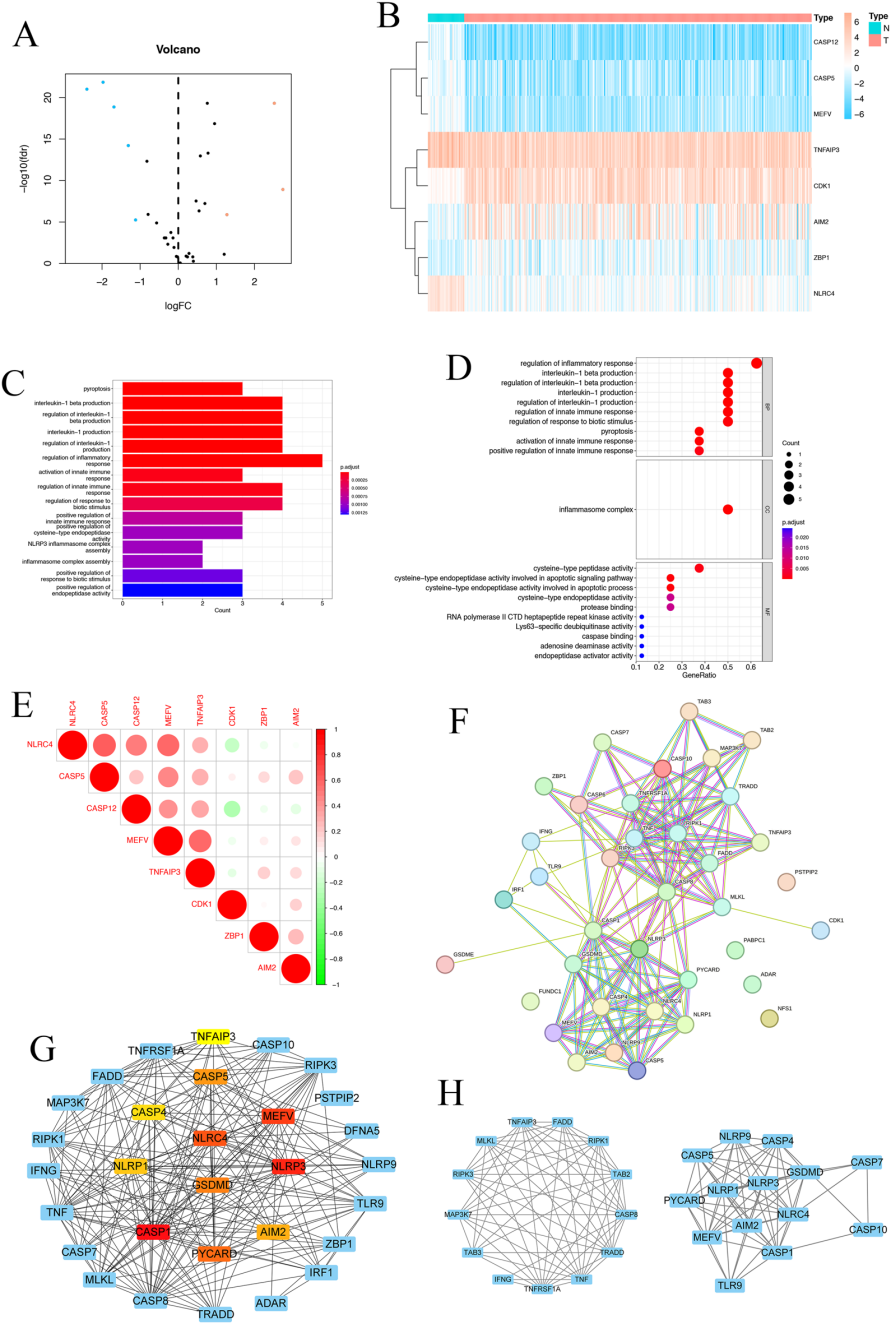
Expression patterns of PANoptosis regulators in LUAD. (**A**) Volcano plot displaying PRGs in LUAD. Blue indicates downregulated genes, and red indicates upregulated genes. The black dot indicates the genes without significant changes. (**B**) Heatmap visualization of the expression levels of PRGs in each sample. "N" represents normal samples, and "T" represents tumor samples. Blue indicates low expression, and red indicates high expression. (**C**) KEGG Enrichment Analysis of PRGs. (**D**) GO Enrichment Analysis of PRGs. (**E**) Spearman correlation analysis of eight differentially expressed PRGs in LUAD. Red circles indicate positive correlations and green circles indicate negative correlations. (**F**) PPI network of the differentially expressed PRGs obtained from the STRING database. (**G**) Top 10 hub genes depicted in a network using “cytohubba” plugin; an increase in the redness of the color, indicates a higher degree value. (**H**) Two modules identified by the “MCODE” plugin.

The 38 PRGs were analyzed for differential expression in LUAD (n = 412) and adjacent normal samples (n = 43). The volcano plot (Fig. 1A) and heatmap (Fig. 1B) showed that eight PRGs were differentially expressed between LUAD and normal adjacent samples. The expression levels of *AIM2*, *CDK1* and *ZBP1* were higher in LUAD tissues, whereas the expression levels of *CASP12*, *CASP5*, *MEFV*, *TNFAIP3*, and *NLRC4* were higher in normal tissues. Next, the genes that exhibited differential expression were examined using GO and KEGG pathway enrichment analyses. These genes were mainly enriched for inflammation, immune function, and cytokine-related biological functions (Fig. 1C,D). Correlations between the eight upregulated and downregulated genes are shown in Fig. 1E. The differentially expressed PRGs were positively correlated, except for *CDK1*, *ZBP1*, and *AIM2* which were negatively correlated with *NLRC4* and *CASP12* expression. *CDK1* was negatively correlated with *MEFV* and *TNFAIP3* expression. The PPI network was constructed using the Cytoscape software and STRING database (Fig. 1F). Finally, the 10 top-ranked hub genes were identified from the PRGs in the network through degree analysis, as presented in Fig. 1G (colored nodes), and two modules were determined using MCODE (Fig. 1H).

### Consensus clustering was used to identify two molecular subtypes

**Figure 2:**
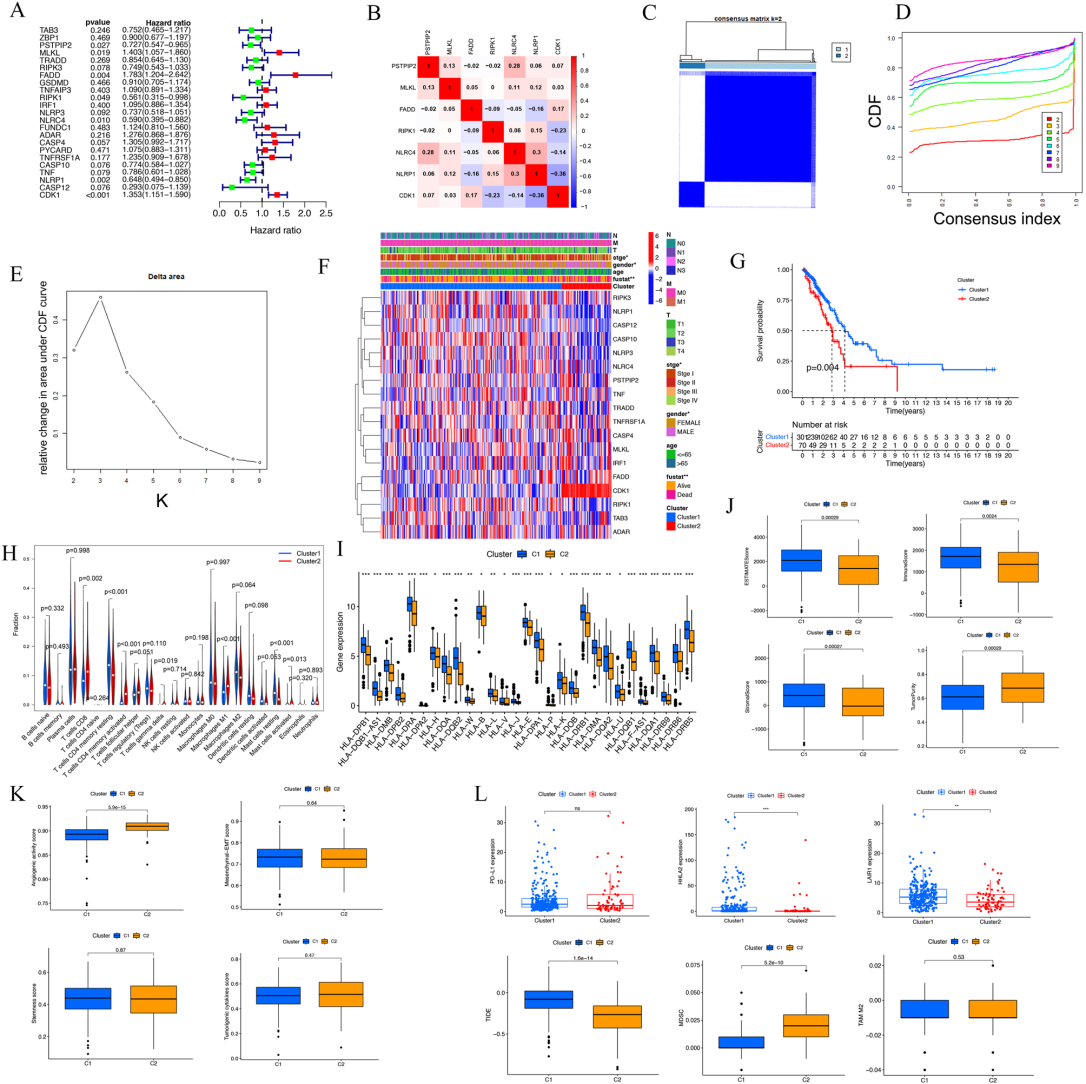
Two clusters on PRGs may predict OS and immune infiltration in LUAD. (**A**) Forest plot of 23 PRGs with univariate Cox regression analysis. (**B**) Correlation of prognosis-related signature genes. (**C**) Correlation between subgroups when the number of clusters k = 2. (**D**) Cumulative distribution function (CDF) when k = 2–9. (**E**) Relative change in area under the CDF curve when k = 2–9. (**F**) Heatmap and clinicopathological features of the two clusters. Blue indicates low expression, and red indicates high expression. (**G**) Comparison of overall survival (OS) between cluster 1 and 2. (**H**) Results of immune cell infiltration using CIBERSOR. (**I**) MHC molecules expression level in two clusters. (**J**) Immune and stromal scores in two clusters. (**K**) Differences of angiogenic activity, EMT, tumorigenic cytokines and stemness scores between cluster 1 and cluster 2. (**L**) Differences of immune escape related markers between cluster 1 and cluster 2. (*P < 0.05; **P < 0.01; ***P < 0.001; ns, not significant).

Univariate Cox regression analyses showed that *PSTPIP2, RIPK1, NLRC4*, and *NLRP1* were protective factors, whereas *MLKL, FADD*, and *CDK1* were risk factors (Fig. 2A). In addition, correlation analyses demonstrated that majority of genes have associations with each other (Fig. 2B). To further investigate the clinical relevance of PRGs, LUAD patients were clustered into subgroups based on gene expression. According to the similarity of PRGs, k = 2 provided the optimal clustering, and the LUAD patients were subsequently separated into two distinct and non-overlapping groups (Fig. 2C–E). Subsequently, we evaluated the presence of significant differences in OS, age, stage, and gender between the two clusters. The results suggested that the prognosis in cluster 1 was substantially (*P* = 0.004) better than in cluster 2 (Fig. 2G). In addition, cluster 1 had lower tumor stages (Fig. 2F) than cluster 2. In conclusion, consensus clustering revealed a significant relationship between PRG expression patterns and clinical parameters.

We analyzed the differences in the immune infiltration characteristics between the two clusters. The CIBERSORT algorithm suggested that cluster 1 had higher levels of activated memory CD4^+^ T cells, CD8^+^ T cells, activated mast cells, and M1 macrophages. M1 macrophages were associated with increased immune cell infiltration (Fig. 2H). In addition, cluster 1 correlated with increased expression of MHC molecules (Fig. 2I). The results from the ESTIMATE algorithm suggested that cluster 2 had lower immune scores, ESTIMATE scores, stromal scores, and higher tumor purities (Fig. 2J) than cluster 1. There were no significant differences between mesenchymal EMT, stemness scores, and tumorigenic cytokines, but angiogenic activity was significantly higher in cluster 2 (Fig. 2K). In addition, the correlation between tumor immune evaluation and PRG expression levels were evaluated to identify cluster-specific differences in immune infiltration. The HHLA2 and LAIR1 immune checkpoints had higher expressions in Cluster 1 (Fig. 2L, upper panel). In addition, cluster 1 had a higher TIDE score and a lower MDSC score than cluster 2 (Fig. 2L, lower panel).

### Prognostic model construction and validation

**Figure 3:**
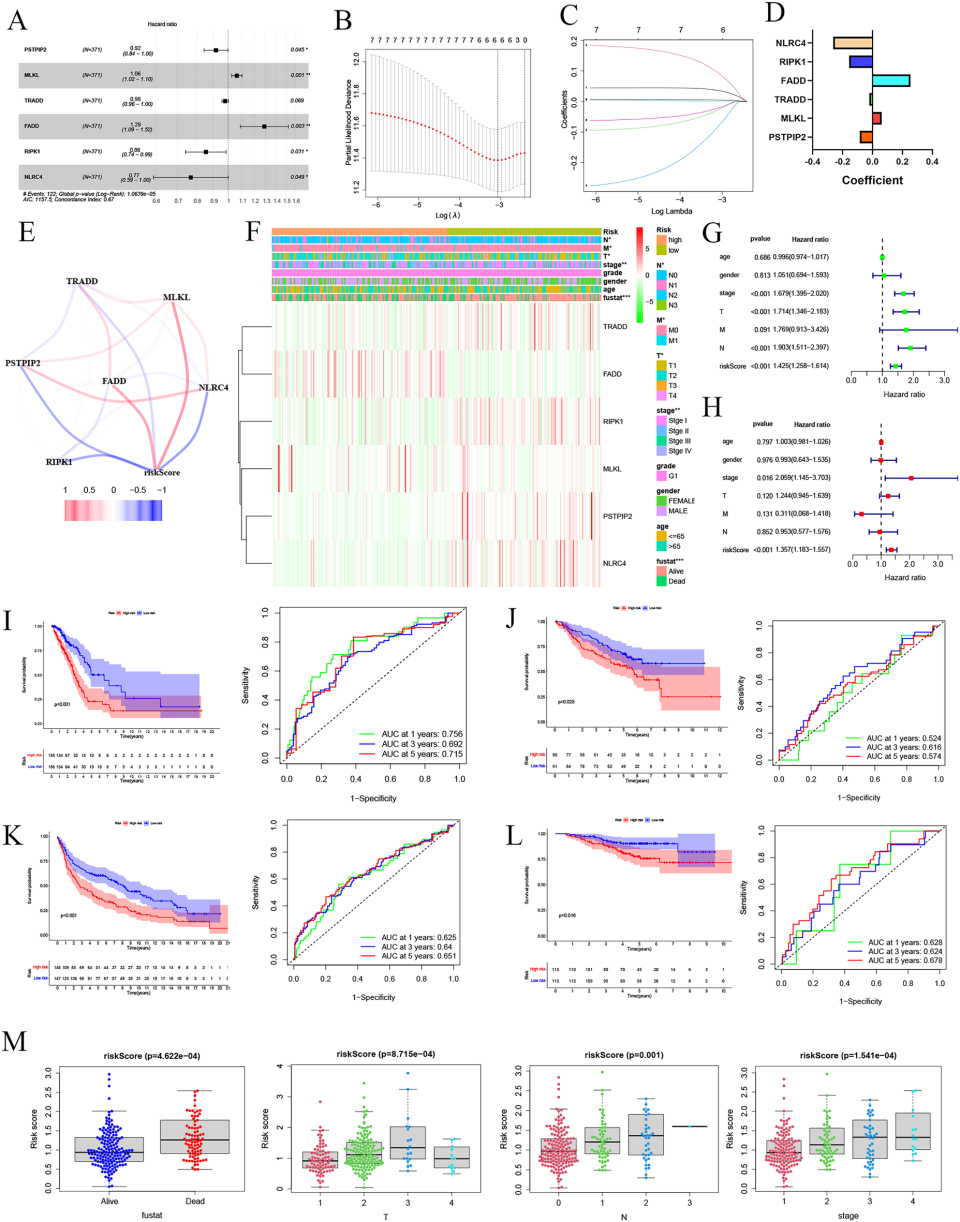
Establishment of a prognostic risk model based on PRGs. (**A**) Univariate Cox regression analysis of PRGs. (**B**,**C**) Constructing the signature using absolute shrinkage and selection operator (LASSO) Cox regression. (**D**) Coefficients of the six genes included in the signature. (**E**) Correlations between the six genes. (**F**) Heatmap showing clinicopathological features' distribution and the expression of the six PRGs in high-and low-risk populations. (**G**) Univariate Cox regression analysis of clinicopathological parameters and OS. (**H**) Multivariate Cox regression analysis of clinicopathological parameters and OS. (**I**) Survival curve and ROC curve analysis of the TCGA cohort. (**J**) Survival curve and ROC curve analysis of the GSE50081 dataset. (**K**) Survival curve and ROC curve analysis of the GSE31201 dataset. (**L**) Survival curve and ROC curve analysis of the GSE30219 dataset. (**M**) Differences in risk scores between subgroups with different clinicopathological parameters. (*P < 0.05; **P < 0.01; ***P < 0.001; ns, not significant).

Based on the correlation between PANoptosis regulators and OS of LUAD patients, univariate Cox regression analysis on the expression levels of 38 PRGs was performed to investigate their clinical relevance. As shown in Fig. 3A, *MLKL* and *FADD* were risk genes with HR > 1, whereas *PSTPIP2*, *TRADD, RIPK1*, and *NLRC4* were protective genes with HR < 1. LASSO-penalized Cox analysis is commonly employed in multiple regression analysis. It allows for variable selection and regularization, enhancing the predictive capacity and accuracy of the statistical model. This technique is widely used to select the most relevant features in high-dimensional data with limited associations, effectively preventing overfitting. Therefore, this technique efficiently identifies optimal predictive markers and provides a prognostic indicator for clinical outcome prediction. The results indicated six genes with the most significant predictive capabilities (Fig. 3B,C). Using the coefficients from the LASSO algorithm, six optimal genes (*MLKL*, *FADD*, *PSTPIP2*, *TRADD, RIPK1*, and *NLRC4*) were chosen to build the risk model (Fig. 3D). Therefore, the corresponding coefficients risk score = (0.061 × expression value of *MLKL*) + (0.253 × expression value of *FADD*) − (0.086 × expression value of *PSTPIP2*) − (0.021 × expression value of *TRADD*) − (0.156 × expression value of *RIPK1*) − (0.262 × expression value of *NLRC4*). Correlations between the risk score and *NLRC4, RIPK1, FADD, TRADD, MLKL*, and *PSTPIP2* are shown in Fig. 3E.

To examine the prognostic value of these gene signature models, LUAD patients were divided into low- and high-risk groups based on the median risk score. The clinically relevant heatmap displayed the differential expression of six prognostic genes in high- and low-risk groups (Fig. 3F). We found substantial variations in clinical data, such as fustat (*P* < 0.001), stage (*P* < 0.01), and TNM (*P* < 0.05). Cox univariate analysis indicated that risk scores, T stage, and N stage were significantly related with OS in patients with LUAD (Fig. 3G, P < 0.001). Multivariate Cox regression analysis was applied to determine if the risk score was independent of other clinicopathological characteristics as a predictor of LUAD. The results indicated that the risk score was independently associated with OS (Fig. 3H, P < 0.001). In conclusion, our findings suggest that the six-gene risk signatures are reliable predictors of patient prognosis in LUAD, regardless of other clinicopathological characteristics, including histological grade, gender, age, and pathological stage.

### Survival analyses and ROC curve based on prognostic model

Survival analyses were performed on data from 371 patients with LUAD in the TCGA. The results demonstrated that patients with high-risk scores tended to have worse OS than low-risk group (Fig. 3I, P < 0.001). The 5-year OS rates were 49.3% and 22.5% in the low-and high-risk groups, respectively. ROC curve analysis showed that the area under the curve (AUC) at 1-year, 3-year, and 5-year OS were 0.756, 0.692, and 0.715, respectively. These results indicated a high capacity for predicting survival outcomes. In addition, the risk score distribution of patients with LUAD was plotted, and their survival status was visualized using a dot matrix (Fig. S1).

Then, we utilized three GEO datasets to assess the predictive ability of our risk model in these validation cohorts. Based on the cutoff value determined from the TCGA cohort, the patients in these cohorts were divided into low-risk and high-risk groups. Survival analysis results showed that the low-risk group had significantly increased OS than the high-risk group (Fig. 3J–L, left). This improved OS corresponded with the results of the TCGA cohort. The AUC for 1-year OS was 0.524–0.628, 3-year OS was 0.616–0.64, and 5-year OS was 0.574–0.678. These results validated the predictive performance of the risk model (Fig. 3J–L, right). We analyzed differences in risk scores between subgroups based on various clinicopathological factors. The results proved that patients with stage II–IV, N1–3, and T2–3 tumors had higher risk scores. Therefore, we concluded that higher risk scores correlate with advanced tumor staging (Fig. 3M).

### Construction of a nomogram

**Figure 4:**
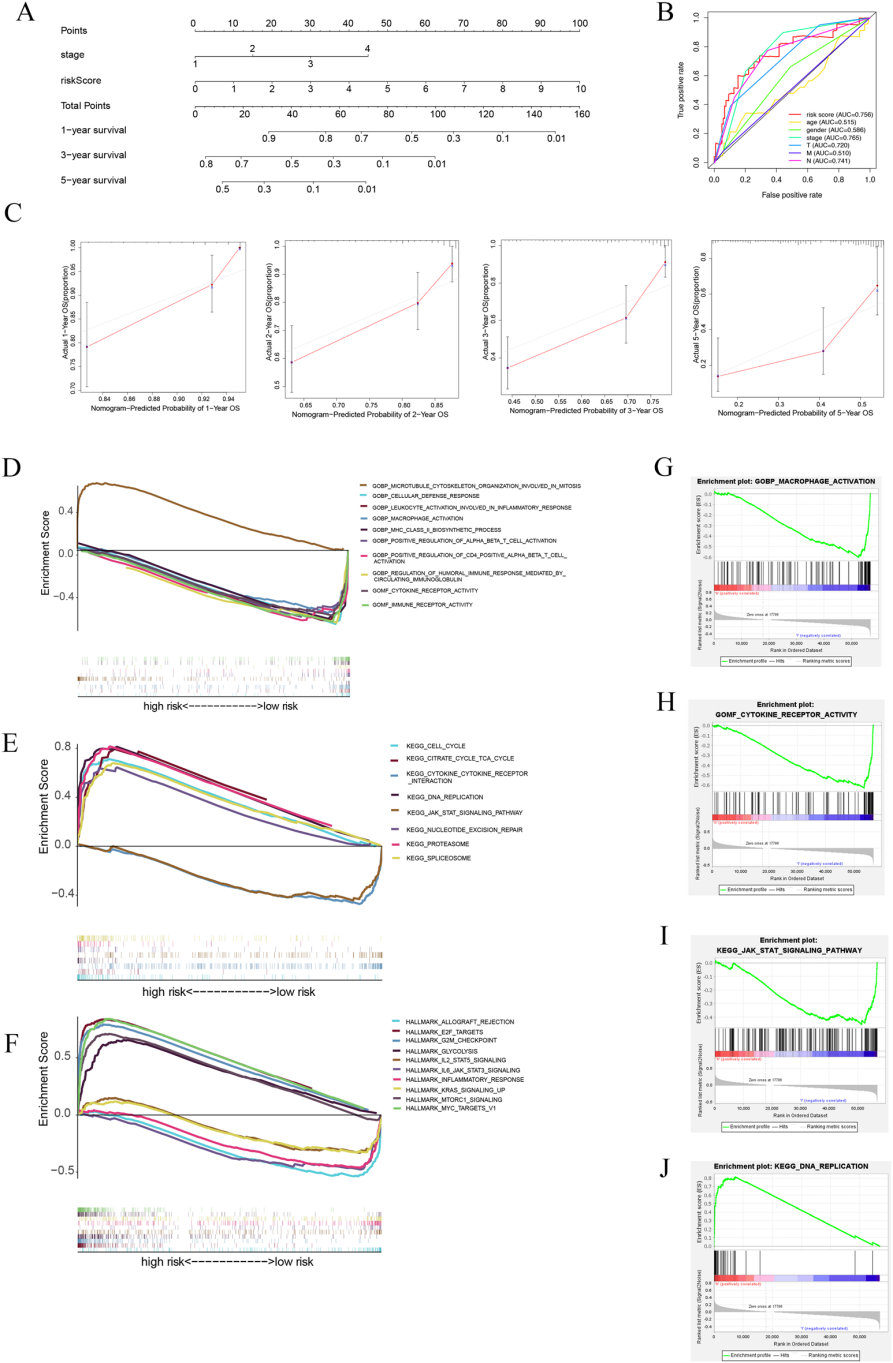
Construction of the nomogram and the functional enrichment analysis for six Genes. (**A**) 1-, 3-, and 5-year OS in BC patients can be systematically predicted by combining clinical data with prognostic nomograms. (**B**) Analysis of the predictive performance of the model using the ROC curve. (**C**) Calibration plots of the nomogram for predicting the probability of 1-, 2-, 3- and 5-year survival. (**D**–**F**) GO, KEGG and HALLMARK analyses by GSEA. (**G**) Macrophage activation is higher in the low-risk group. (**H**) Cytokine receptor activity is higher in the low-risk group. (**I**) JAK-STAT signaling pathway activation is higher in the low-risk group. (**J**) DNA replication is higher in the high-risk group.

To promote clinical application of the risk model, a comprehensive prognostic nomogram based on tumor stage and risk score was developed (Fig. 4A). The nomogram accurately predicted the 1-, 3-, and 5-year OS for LUAD patients. The AUC for the risk model was 0.756, demonstrating a close relationship between the prediction performance of the tumor stage and AUC (Fig. 4B). The results proved that the model had an excellent predictive value for patients with LUAD. Calibration plots demonstrated that the actual 1-, 2-, 3-, and 5-year survival rates closely matched the predicted survival rates (Fig. 4C).

### Gene set enrichment analysis

We performed KEGG (Fig. 4D), GO (Fig. 4E), and HALLMARK (Fig. 4F) enrichment analyses to identify differentially expressed genes between the two risk groups and to investigate subgroup outcomes in regard to related biological processes and pathways. The results showed that immune response and inflammation-related biological functions associated with low-risk groups included “Inflammatory response,” “Cytokine receptor activity,” “Cytokine- cytokine receptor interaction,” “JAK-STAT pathway,” “Macrophage activation,” and “Cytokine receptor activity.” Tumor protective functions such as “DNA replication,” “MYC targets,” “Nucleotide excision repair,” “Cell cycle,” “Microtubule cytoskeleton organization involved mitosis,” and “Spliceosome” were activated in the high-risk group and predicted their poor prognosis. Partial results of the enrichment analyses are shown in Fig. 4G–J.

### Immune infiltration and malignant features correlate with prognosis-related PRGs

**Figure 5:**
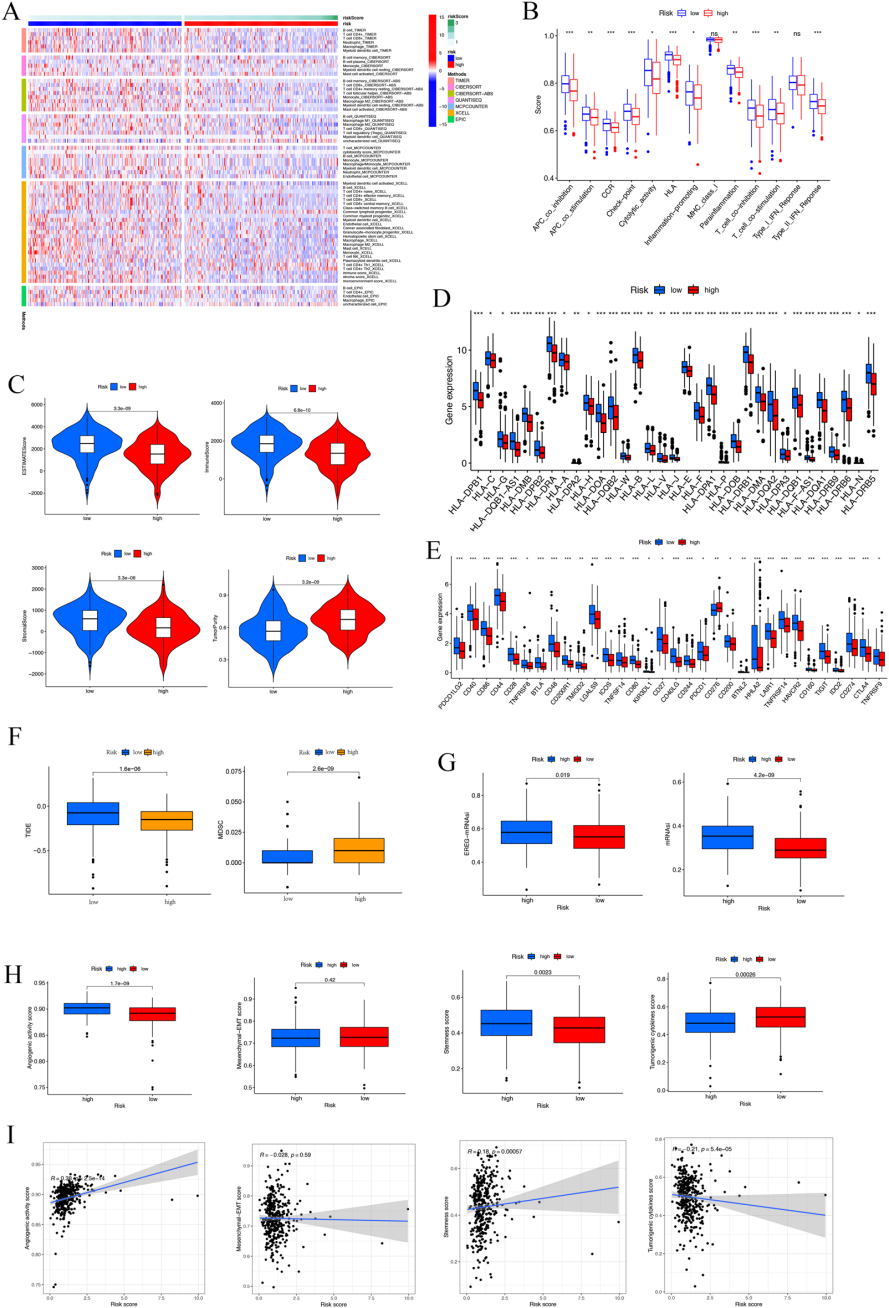
Association among prognosis-related PRGs, immune infiltration, and malignant features. (**A**) Correlation between risk score and altered immune landscape. Heatmap for anticancer immunity cycles pattern. (**B**) ssGSEA used to correlate immune cell subsets and related functions. (**C**) Immune and stromal scores. (**D**) MHC molecules expression level. (**E**) Immune checkpoint expressions in high and low-risk patients. (**F**) TIDE score and MDSC level between the high-and low-risk groups. (**G**) Differences in TSIs between the two groups. (**H**) Differences in angiogenic activity, mesenchymal-EMT, tumorigenic cytokines and stemness scores between the high-and low-risk groups. (**I**) Correlation between the risk score and angiogenic activity, mesenchymal-EMT, tumorigenic cytokines and stemness scores. (*P < 0.05; **P < 0.01; ***P < 0.001; ns, not significant).

Functional enrichment analysis revealed that the functions of PRGs primarily include inflammation, DNA proliferation, and immune response. To validate these results, an immune infiltration analysis was performed. Using the MCPCOUNTER, CIBERSORT, TIMER, QUANTISEQ, and other algorithms, the expression level of activated natural killer (NK) cells, memory B cells, CD4^+^ T cells, M2 macrophages, and monocytes was found to be lower in the high-risk group than in the low-risk group (Figs. 5A, S2). The reduced expression of these factors indicates that immune infiltration could have impacted the prognosis of the patients. In addition, in the correlation analysis, high-risk scores were negatively associated with antitumor immune cells (Fig. S3). Furthermore, quantifying enrichment fractions suggested that the prognosis in high-risk groups may be influenced by lower immune function, including antigen presenting cell cell, chemokine receptor, and inflammation functions (Fig. 5B). Similarly, the ESTIMATE algorithm identified lower ESTIMATE, immune, and stromal scores as well as higher tumor purities in the high-risk group (Fig. 5C). The expression of MHC molecules was substantially increased in the low-risk group (Fig. 5D). Furthermore, a clear correlation between low-risk patients and an increased expression of various immune checkpoints, such as CTLA4, HAVCR2, PD-1, and PD-L1, was identified (Fig. 5E). These patients may benefit from immune checkpoint inhibitors. Interestingly, the low-risk group had higher TIDE scores, lower MDSC levels (Fig. 5F), and lower TSIs, including EREG-mDNAsi and mDNAsi (Fig. 5G). The high-risk group had higher angiogenic activity and stemness scores and lower tumorigenic cytokine scores (Fig. 5H). In addition, the link between the risk score and the four malignant features (Fig. 5I) suggested that the risk score positively correlated with the angiogenic (R = 0.38, *P* < 0.001) and stemness scores (R = 0.18, *P* < 0.001).

### Signature comparison of somatic mutation and TMB

**Figure 6:**
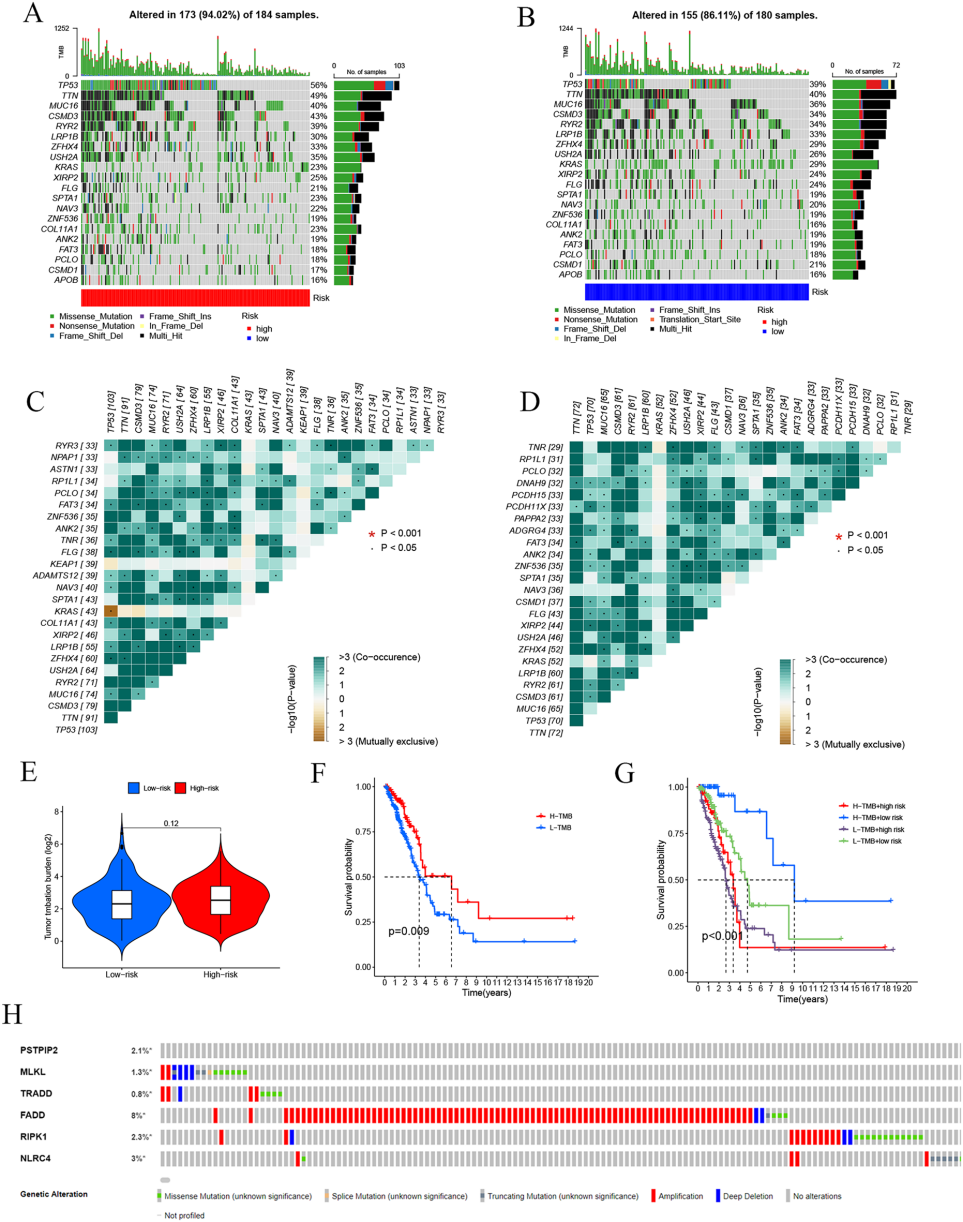
Comparison of somatic mutation and TMB in the six PRGs. (**A**,**B**) Waterfall maps of the somatic mutations in two risk groups. (**C**,**D**) Heatmap of co-occurrence and mutually exclusive mutations of the differently mutated genes in the high-risk group and the low-risk group. *P < 0.001. (**E**) Comparison of TMB between the two risk groups. (**F**) Survival difference between groups with high and low TMB levels. (**G**) Difference in overall survival according to TMB and risk score. (**H**) Mutation rates of six genes (*PSTPIP2, MLKL, TRADD, FADD, RIPK1,* and *NLRC4*) in patients with LUAD from the cBioPortal database. (*P < 0.05; **P < 0.01; ***P < 0.001; ns, not significant).

We downloaded basic nucleotide variation data from the TCGA to explore the differences in genomic mutations between the two risk groups. *TP53* (56%), *TTN* (49%), *CSMD3* (43%), *MUC16* (40%), and *RYR2* (39%) were the top five genes with the highest mutation frequencies in the high-risk group (Fig. 6A). In contrast, *TTN* (40%), *TP53* (39%), *MUC16* (36%), *CSMD3* (34%), and *RYR2* (34%) were the top five genes in the low-risk group (Fig. 6B). The high-risk group had a higher mutation rate than the low-risk group. The interactions of somatic mutations were also investigated. Gene mutation co-occurrence existed in almost all of the genes, whereas mutually exclusive *TP53-KRAS* mutations were found in the high-risk group (Fig. 6C). Gene mutation co-occurrence had a decreased prevalence in the low-risk group (Fig. 6D). TMB, an emerging biomarker, refers to the number of mutated bases per million bases in tumor tissue. It is increasingly utilized for the prediction of patient prognosis. There were no significant differences in TMB between the two groups (Fig. 6E). Among patients with LUAD, the high-TMB group had shorter survival times than the low-TMB group (Fig. 6F). The prognosis was significantly more increased in the low-risk and low TMB groups than in the other groups (Fig. 6G). This improvement in prognosis indicated that our prediction model optimized the prediction performance of TMB. Finally, the following mutation rates were detected (Fig. 6H): *PSTPIP2* (2.1%), *MLKL* (1.3%), *TRADD* (0.8%), *FADD* (8%), *RIPK1* (2.3%), and *NLRC4* (3%).

### Potential drug prediction

**Figure 7:**
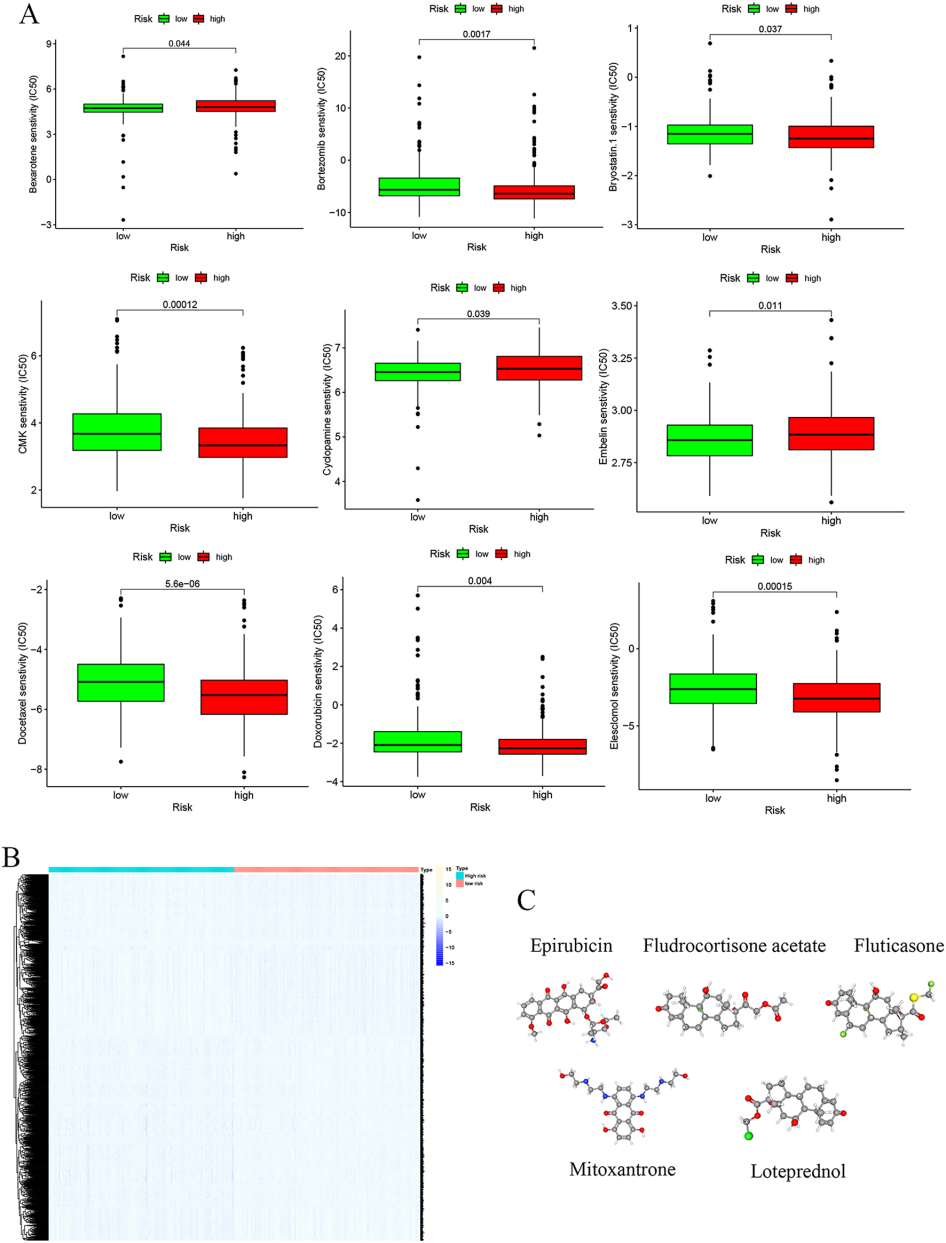
Potential treatment for patients with LUAD based on six PRGs. (**A**) Chemotherapy response for patients with LUAD. (**B**) Differentially expressed genes between the high- and low-risk groups. (**C**) The 3D structure of five potential small-molecule drugs screened from the cMap database. *P < 0.05; **P < 0.01; ***P < 0.001; ns, not significant.

Sensitivity analysis of chemotherapeutic drugs showed that bortezomib, bryostatin 1, CMK, docetaxel, doxorubicin, and elesclomol were more effective in the low-risk groups, whereas bexarotene, cyclopamine, and embelin were more efficacious in the high-risk groups (Fig. 7A). The differentially expressed genes in the two risk groups were categorized as either up-regulated or down-regulated (Fig. 7B). These genes were submitted to the CMAP database to seek small-molecule drug candidates for the treatment of LUAD. Using FDR < 0.05 and a standardized score as criteria for screening, 10 small-molecule compounds with a treatment effect on LUAD (a negative enrichment score represents the inhibitory effect) were identified (Table 1). The 3D structures of the top five small-molecule drugs (Fig. 7C) are displayed in the PubChem database.

**Table 1:** The ten small molecule drugs of the CMP dataset.

Compound name	Mechenism of activities	FDR value	Standardized score
Fluticasone	Glucocorticoid receptor agonist	< 0.05	− 1.7593
Fludrocortisone-acetate	Glucocorticoid receptor agonist	< 0.05	− 1.6844
Mitoxantrone	Topoisomerase inhibitor	< 0.05	− 1.662
Epirubicin	Topoisomerase inhibitor	< 0.05	− 1.6536
Loteprednol	Glucocorticoid receptor agonist	< 0.05	− 1.6405
CAY-10585	HIF modulator	< 0.05	− 1.6394
Niclosamide	STAT inhibitor	< 0.05	− 1.6346
Flunisolide	Cytochrome P450 inhibitor	< 0.05	− 1.6312
BRD-K99615199	Progesterone receptor agonist	< 0.05	− 1.6277
Ursolic-acid	Caspase inhibitor	< 0.05	− 1.619

### Immunochemistry validation

**Figure 8:**
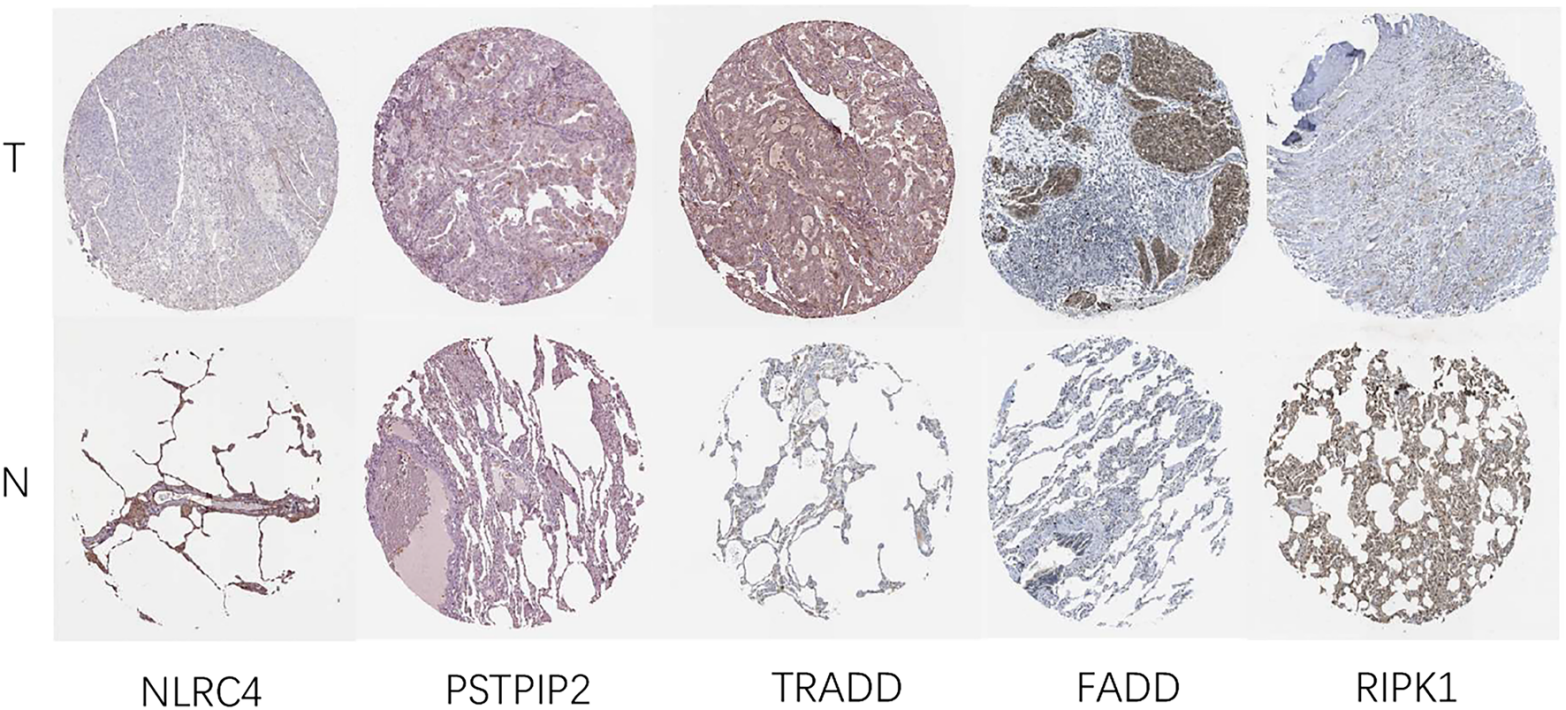
Protein expression of five prognostic genes using the HPA Database.

As shown in Fig. 8, the expression of PRG-related proteins, such as *NLRC4, FADD, TRADD*, and *RIPK1*, was lower in LUAD tissue than in normal lung tissue. PSTPIP2 was not evident in either tissue type. MLKL was not included in Fig. 8 since it was not included in the HPA database.

### Verified expression of six mRNA in lung cancer cell lines using qRT-PCR

**Figure 9:**
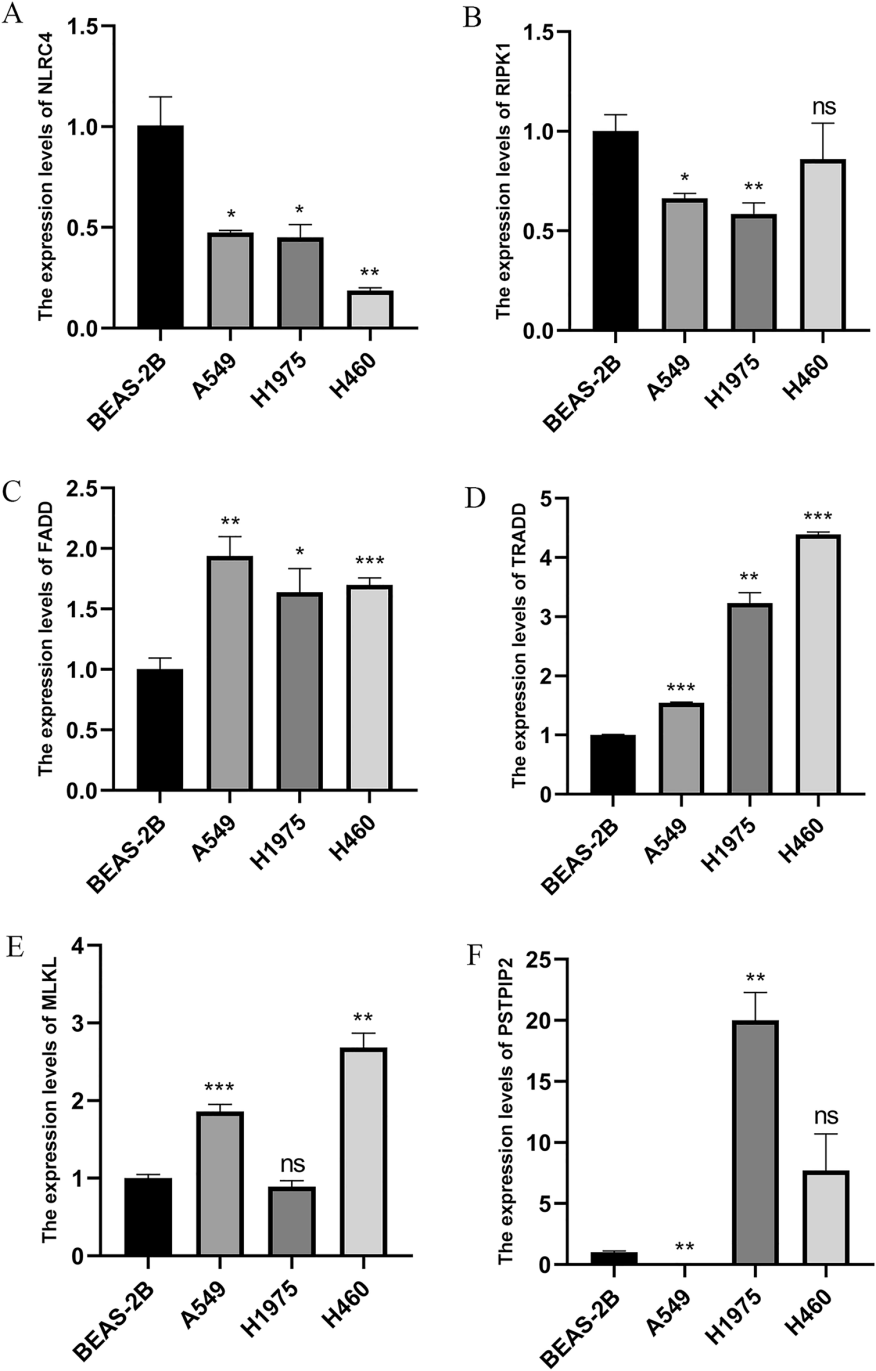
Results of qRT-PCR analysis. (**A**) NLRC4. (**B**) RIPK1. (**C**) FADD. (**D**) TRADD. (**E**) MLKL. (**F**) PSTPIP2. Data are shown as the mean ± S.D. (*P < 0.05; **P < 0.01; ***P < 0.001; ns, not significant).

Compared with BEAS-2B cells, the expression levels of *NLRC4* were statistically lower in A549, H460, and H1975 cells (Fig. 9A). The expression levels of *RIPK1* were also significantly lower in A549 and H1975 cells (Fig. 9B). *FADD* and *TRADD* were highly expressed in A549, H460, and H1975 cells (Fig. 9C,D). *MLKL* expression was higher in A549 and H460 by contrast with BEAS-2B cells (Fig. 9E), and the expression of *PSTPIP2* was very low in A549, but high in H1975 (Fig. 9F). These results validated the differential expression of the six PRGs in normal and lung cancer samples, demonstrating their potential as predictive signatures.

## Discussion

As a newly discovered mechanism of cell death, PANoptosis has piqued the interest of academics. Currently, research on PANoptosis is focused on infections. The innate immune system recognizes conserved molecules, such as Z-RNA and Z-DNA, in pathogens and initiates the activation of pattern recognition receptors, binding of inflammatory agents, passage of death domain-containing receptors, and signaling for the highly interconnected process of PANoptosis^55^. Microbial infections have specific sensors, such as ZBP-1 binding influenza A virus ribonucleoproteins, among other inflammatory factors, which form the ZBP-1 dependent PANoptosome^56^. *Yersinia* suppresses the host protein Transforming growth factor-βactivated kinase-1 (TAK1). Cells deficient in TAK1 form a complex consisting of RIPK1, CASP8, ASC, and NLRP3, thereby activating downstream CASP3/7, phosphorylating Mixed lineage kinase domain-like protein (MLKL), cleaving GSDMD, and ultimately leading to RIPK1-dependent PANoptosis^57^. The link between PANoptosis and tumors is currently being explored, and recent studies have identified tumor-associated markers of PANoptosis. Investigators have found that loss of Cysteine desulfurase (NFS1) significantly enhances the sensitivity of Colorectal cancer (CRC) cells to oxaliplatin, triggering PANoptosis through increased intracellular reactive oxygen species levels^58^. Interferon regulatory factor 1 (IRF1) has also been characterized as a PANoptosis upstream regulator, and the attenuated cell death in CRC in IRF1-knockdown mice is a consequence of defective PANoptosis^59^. While the oncological investigation of PANoptosis remains relatively limited, the utilization of bioinformatic analysis holds the potential to provide valuable insights for forthcoming basic and clinical research endeavors.

In this research, we have pinpointed six PRGs associated with the prognosis of LUAD: NLRC4, FADD, TRADD, RIPK1, PSTPIP2, and MLKL. Our qRT-PCR analysis has convincingly revealed their distinct expression patterns within LUAD cell lines. We used KEGG and GO enrichment analyses to explore the potential biological functions of PRGs in LUAD. The training set (TCGA cohort) was used to develop the prognostic model, while the validation set (GEO cohort) was used to evaluate the model's reliability. We computed the risk score using the PRG model, and it autonomously forecasted the outcomes for patients with LUAD. Additionally, we developed prognostic nomograms incorporating both clinical characteristics and risk scores. The risk signature identified in this study could assist clinicians in making accurate, personalized survival predictions. Additionally, our research revealed a strong correlation between PRGs, somatic mutations, and the immune microenvironment in LUAD. Our risk model can also predict the extent of immune infiltration in LUAD patients, and we have identified potential personalized drug options for clinical application.

In this study, *NLRC4*, *RIPK1*, *TRADD*, and *PSTPIP2* showed elevated expressions in patients with LUAD and were associated with improved prognosis, whereas *MLKL* and *FADD* showed elevated expressions but were associated with poor prognosis. NLRC4 is a well-known and essential factor in PANoptosis that involves pyroptosisand apoptosis. The NLRP3 and NLRC4 inflammasomes induce Poly (ADP-Ribose) Polymerase 1 cleavage in both apoptotic and pyroptotic pathways^59^. In addition, the NLRC4 inflammasome recruits CASP8 by interacting with the death effector domain of CASP8 and subsequently mediates cellular PANoptosis^60^. Furthermore, investigators have demonstrated the inhibitory effect of NLR inflammasomes in colitis-associated cancer using an animal model lacking the NLRC4 inflammasome components^61^. RIPK1, a multidomain protein that includes an intermediate domain, an N-terminal kinase domain, and a C-terminal death domain, is a key factor in necroptosis^62^. RIPK1 mediates the activation of CASP8, RIPK3, MLKL, and NLRC3 to initiate PANoptosis under conditions of TAK1 deficiency. RIPK1 regulates apoptosis and necroptosis depending on its activity, phosphorylation, or ubiquitination status. Elevated RIPK1 expression significantly leads to cisplatin-induced apoptosis in human esophageal cancer cells^63^. PSTPIP2 is an F-BAR protein and is primarily expressed in macrophages, where it coordinates actin’s function in the cytoskeleton^64^. PSTPIP2 is an inflammatory suppressor under usual circumstances. Loss of PSTPIP2 exacerbates chronic recurrent multifocal osteomyelitis and sepsis^64^. In our results, PSTPIP2 expression was low in the high-risk group; we speculate that low levels of PSTPIP2 aggravate systemic inflammation in patients with LUAD, thereby reducing their survival time. MLKL was recognized as the final effector of necroptosis and a downstream target of RIPK3. Therefore, MLKL may have pro- and anti-cancer roles in different tumors^65^. Studies have found that when MLKL phosphorylation levels are elevated, patients with colon and esophageal cancers have poor prognoses and survival outcomes^66^. Meanwhile, low MLKL expression is linked to poor prognoses in cervical and ovarian cancers^67,68^. Our evidence suggests that MLKL acts as a cancer promoter in LUAD, and it may mediate the initiation of inflammation in the tumor microenvironment, thereby promoting tumor metastasis and growth. MLKL, along with RIPK1 and RIPK3, induce neutrophil extracellular traps, allowing them to act as a physical barrier to protect tumor cells from T or NK cell-mediated cytotoxicity^69^. FADD participates in and regulates most signalosome complexes, including the PANoptosome, FADDosome, inflammasome, and necrosome complexes^70^. FADD recruits regulatory proteins of the NF-κB and MAPK pathways, thereby promoting proliferation and the cell cycle^71^, and our results and HPA data demonstrate that indicating that FADD is a poor prognostic marker of lung cancer. In the somatic mutation analysis, FADD was mutated at a higher frequency (8%) than the other five genes, dominated by amplification. The literature indicates that FADD is located on chromosome 11q13.3, and the amplification of this region is a common finding in human tumors and is related with a poor prognosis^72,73^. TRADD is an adaptor for apoptosis mediated by TNFR1 and NF-κB activation^74^. TRADD deficiency in mice accelerates tumor formation in a model of chemically induced carcinogenesis. In in vitro experiments, primary cells lacking TRADD show reduced accumulations of P19 (ARF) tumor suppressor proteins. This reduction is a consequence of the dynamic shuttling of TRADD from the cytoplasm into the nucleus, which regulates the interaction between P19 (ARF) and its E3 ubiquitin ligases, thereby inhibiting tumor growth^74^. Patients with acute myeloid leukemia who have high TRADD expression show a significantly prolonged OS similar to that of patients with LUAD^75^. In conclusion, our study has identified six Prognostic-Related Genes (PRGs) that are significantly associated with the prognosis of patients with Lung Adenocarcinoma (LUAD). These PRGs serve as valuable reference points for the advancement of biomarker development and the design of pharmaceutical interventions.

In the low-risk group, the quantity of activated NK cells, memory B cells, and CD4+ T cells, along with the expression levels of immune function markers and human leukocyte antigen molecules, was significantly higher when compared to the high-risk group. This indicates that the process of PANoptosis has an inseparable link with immune infiltration. For example, the cytokine interleukin (IL)-1β is an end product of PANoptosis. The IL-1 signaling cascade activates dendritic cells and macrophages and regulates the T helper (Th)-1/Th17 differentiation of CD4^+^ T cells and CD8^+^ T cell effector functions^76,77^. NLRC4 activation is critical for cytokine and chemokine production in tumor-associated macrophages and is required to generate CD4^+^ and CD8^+^ T cells in the B16F10 melanoma mouse model^78^. Notably, the TIDE score and immune checkpoint marker expressions were higher in the low-risk group than in the low-risk group. This indicates that the antitumor effect of immune cells was inhibited in this group, that the effect of high tumor immune infiltration was not exerted fully, and that the use of immune checkpoint inhibitors in this group of patients might have promising results. We also found that the risk score could predict the angiogenesis and stemness of LUAD. In addition, we simulated the role of drugs in various reactions, demonstrating that the sensitivity to multiple drugs differed significantly between high-risk and low-risk groups. We predicted 10 potential therapeutic compounds for patients with LUAD By comparing the differentially expressed genes between the high- and low-risk groups.

Currently, the majority of advanced-stage LUAD patients require chemotherapy^79^. However, defects in the apoptotic mechanisms of cancer cells are associated with multi-drug resistance (MDR)^80^. Therefore, it is crucial to use risk scoring system to identify patients who may be sensitive to relevant drugs. Our results demonstrate that Bexarotene, Cyclopamine, and Embelin were more efficacious in the high-risk groups. Bexarotene is a synthetic retinoid modulator of retinoid X receptors (RXRs), selectively binding and activating RXRs including RXRα, RXRβ, and RXRγ^81^. It plays a critical role in regulating cell growth, activating apoptosis, and inducing differentiation. It has been found that bexarotene promotes the expression of PPARγ by enhancing Slc10a2, while decreasing the expression of mTOR, thereby promoting cell death in A549 cells^82^. Cyclopamine effectively inhibits xenograft tumors in mice with NSCLC by directly suppressing mitochondrial respiration in lung cancer cells^83^. Embelin, a potent quinone derivative from E. ribes, has been extensively studied due to its anthelmintic, antitumor, anti-inflammatory, antidiabetic, and anticonvulsant properties^84^. Specifically, embelin has been shown to effectively induce apoptosis in A549 cells^85^. Additionally, we have also predicted potential effective compounds for high-risk LUAD patients through database analysis, including anthracycline anticancer drugs (Epirubicin and Mitoxantrone) and hormonal drugs (Loteprednol, Fluticasone, and Fludrocortisone acetate). Our study has revealed the enormous potential of these mentioned drugs to promote PANoptosis in lung adenocarcinoma and prolong patient survival. However, it should be noted that the aforementioned drugs have not been used in the LUAD population, and further in-depth basic research and clinical trials are necessary for validation. In summary, these results suggest that the PANoptosis-based six gene prediction model not only predicts poor prognosis in LUAD patients but also has the potential to assist in tailoring personalized treatment plans based on their expression levels.

There were limitations to our study. First, the study relied on data from public databases; therefore, prospective cohort studies in the real world are necessary to verify the risk score formula. Furthermore, the differential expression of related genes was only confirmed through cellular assays, so the biological mechanisms underlying the effects of PRGs in LUAD remain unclear. Comprehensive in vitro and in vivo experiments are required to investigate the functions of the six LUAD prognostic genes.

## Conclusion

This study identified molecular subtypes based on PRGs in LUAD and constructed a prognostic signature. In addition, the immune infiltration landscape, gene mutation status, and drug prediction of different risk groups were also analyzed. This signature may contribute to the clinical evaluation of prognosis and drug therapy.

### Supplementary Information

Supplementary Figure S1.

Supplementary Figure S2.

Supplementary Figure S3.

Supplementary Table S1.

Supplementary Table S2.

Supplementary Table S3.

Supplementary Table S4.

Supplementary Legends.

## Data Availability

Publicly available datasets were analyzed in this study. This data can be found here: The Cancer Genome Atlas database (https://portal.gdc.cancer.gov/) and Gene Expression Omnibus database (https://www.ncbi.nlm.nih.gov/geo/). All datasets in the present study were downloaded from public databases, including TCGA, and GEO. These public databases allowed researchers to download and analyze public datasets for scientific purposes. The current research follows the TCGA and GEO data access policies and publication guidelines. Users can download relevant data for free, our study is based on open-source data, there are no ethical issues and other conflicts of interest.

## Abbreviations

LUAD: Lung adenocarcinoma
PRGs: PANoptosis-related genes
PSTPIP2: Proline-serine-threonine-phosphatase-interacting protein 2
MLKL: Mixed lineage kinase domain-like protein
TRADD: TNFR1-associated death domain protein
FADD: Fas-associated protein with death domain
RIPK1: Receptor-interacting serine/threonine-protein kinase 1
NLRC4: NLR-family CARD-containing protein 4
PD-1: Programmed cell death 1
PD-L1: Programmed cell death one ligand 1
CTLA4: Cytotoxic T lymphocyte-associated protein 4
ROC: Receiver operating characteristic
