# Supplementary material for: Identifying prognostic genes related PANoptosis in lung adenocarcinoma and developing prediction model based on bioinformatics analysis
Source: Sci Rep. 2023 Oct 20;13:17956. doi: 10.1038/s41598-023-45005-6 (PMC10589340; doi:10.1038/s41598-023-45005-6)
Supplement: Supplementary file 1 — Supplementary Figure S1. [file 41598_2023_45005_MOESM1_ESM.docx]

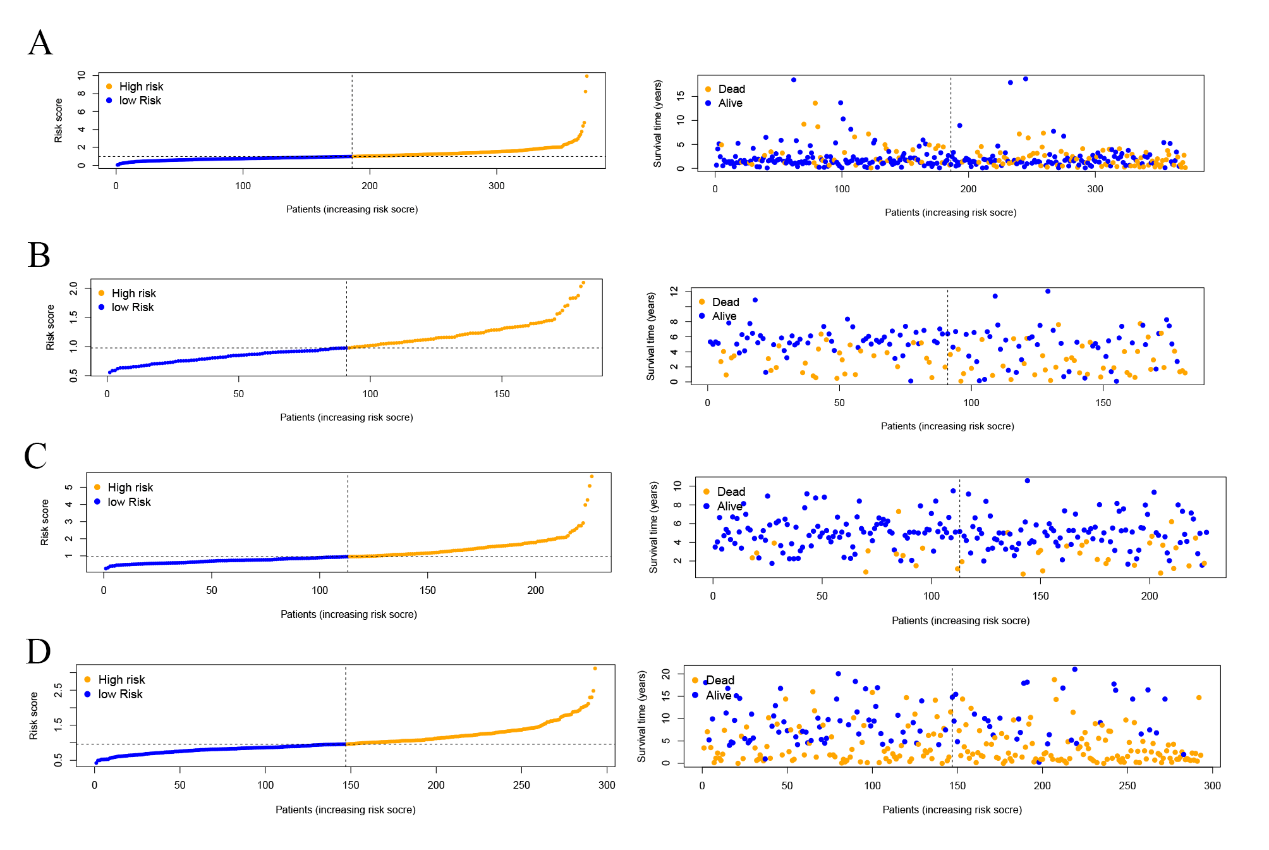


Figure S1. Distribution of risk scores and survival status in the prognostic model. (A) The TCGA cohort. (B) The GSE50081 dataset. (C) The GSE42127 dataset. (D) The GSE31201 dataset. (E) The GSE30219 dataset. (F) The GSE8894 dataset.
