# Supplementary figures and images for: Identifying prognostic genes related PANoptosis in lung adenocarcinoma and developing prediction model based on bioinformatics analysis
Source: Sci Rep. 2023 Oct 20;13:17956. doi: 10.1038/s41598-023-45005-6 (PMC10589340; doi:10.1038/s41598-023-45005-6)

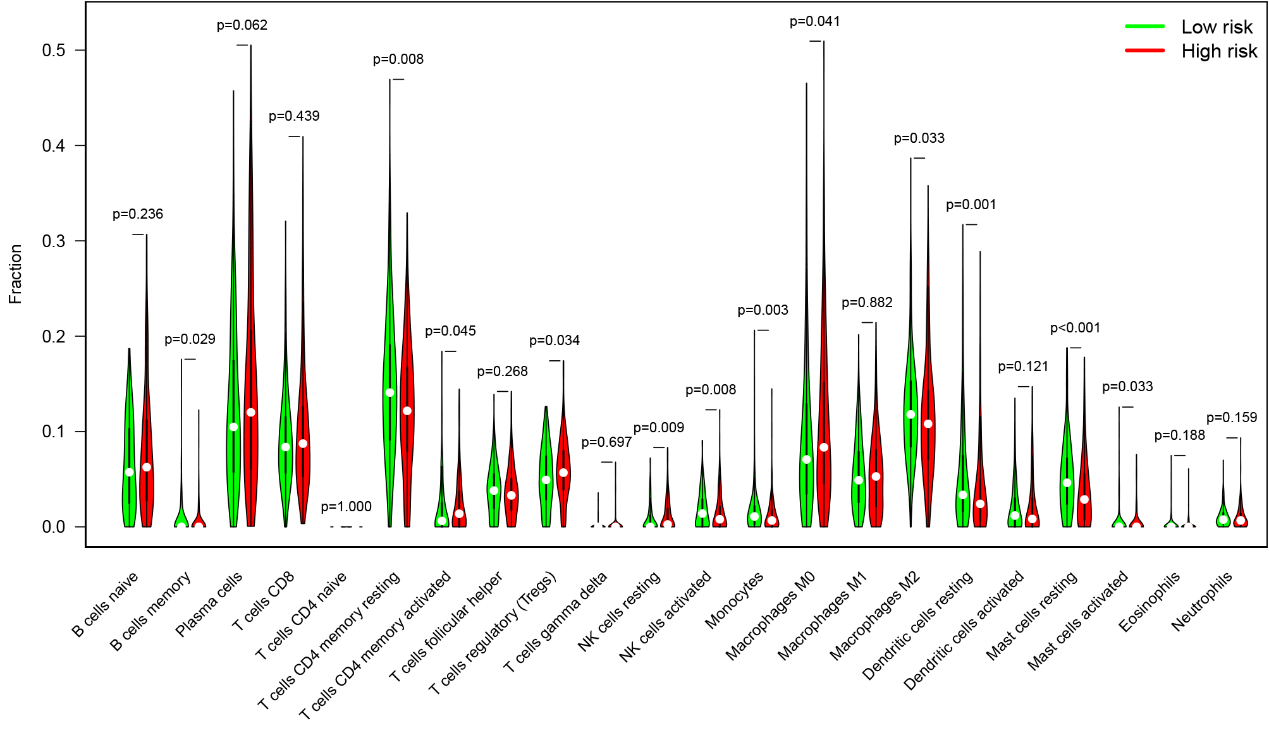


Figure S2. Immune cell infiltration using CIBERSORT between the high- and low-risk groups.

Supplement: Supplementary file 2 — Supplementary Figure S2. [file 41598_2023_45005_MOESM2_ESM.docx]

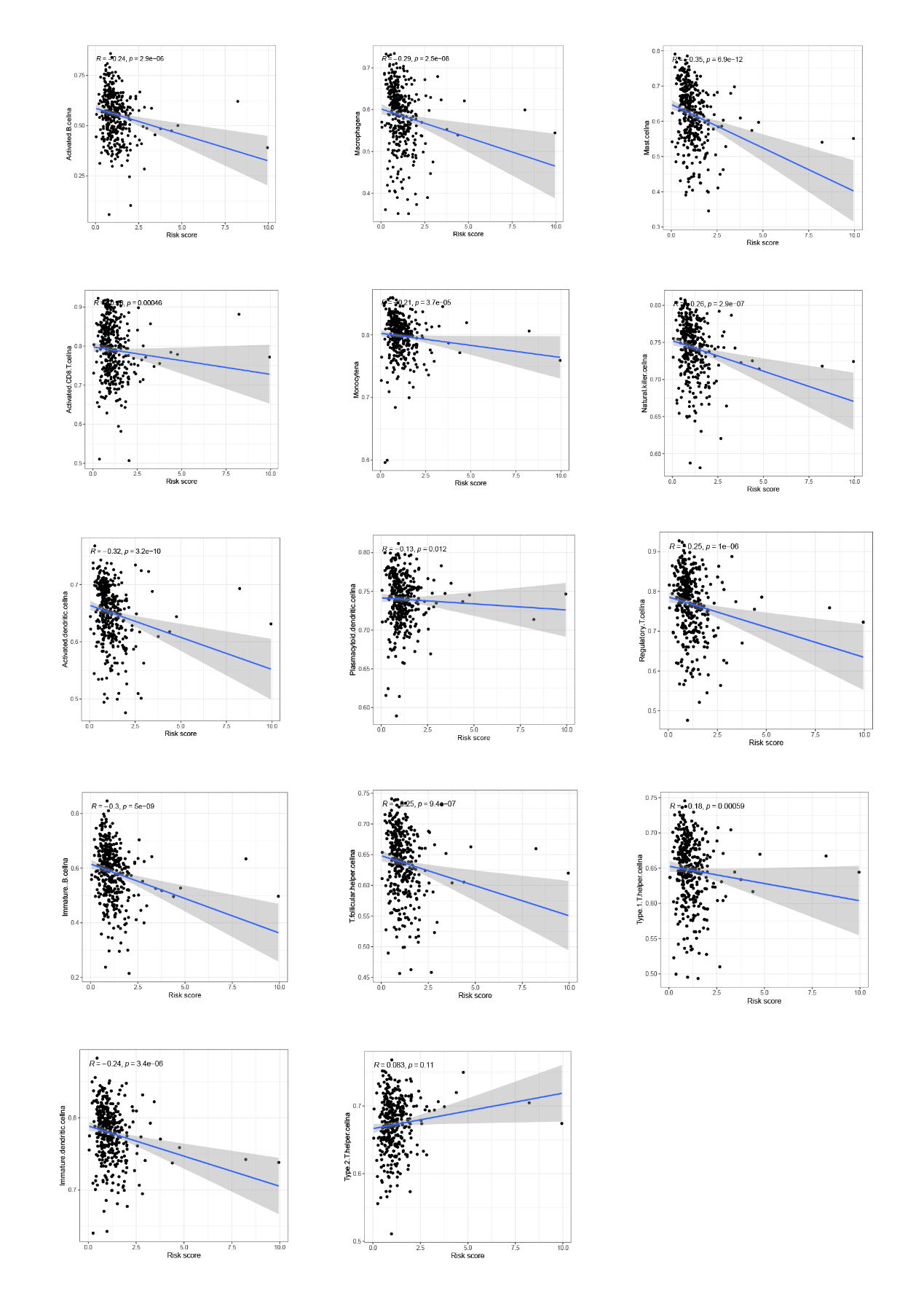


Figure S3. Relationship between risk score and the proportion of immune infiltration.

Supplement: Supplementary file 3 — Supplementary Figure S3. [file 41598_2023_45005_MOESM3_ESM.docx]
