# Supplementary material for: Identifying prognostic genes related PANoptosis in lung adenocarcinoma and developing prediction model based on bioinformatics analysis
Source: Sci Rep. 2023 Oct 20;13:17956. doi: 10.1038/s41598-023-45005-6 (PMC10589340; doi:10.1038/s41598-023-45005-6)
Supplement: Supplementary file 8 — Supplementary Legends. [file 41598_2023_45005_MOESM8_ESM.docx]

**Supplementary Materials**

Table S1 Clinical information in TCGA

Table S2 Gene expression matrix

Table S3. Gene sets

Table S4. Primers of genes

Figure S1. Distribution of risk scores and survival status in the prognostic model. (A) The TCGA cohort. (B) The GSE50081 dataset. (C) The GSE42127 dataset. (D) The GSE31201 dataset. (E) The GSE30219 dataset. (F) The GSE8894 dataset

Figure S2. Immune cell infiltration using CIBERSORT between the high- and low-risk groups

Figure S3. Relationship between risk score and the proportion of immune infiltration
